# COVID-19 staycations and the implications for leisure travel

**DOI:** 10.1016/j.heliyon.2022.e10867

**Published:** 2022-10-03

**Authors:** Babajide Abubakr Muritala, Ana-Beatriz Hernández-Lara, Maria-Victoria Sánchez-Rebull

**Affiliations:** Departament de Gestió d'Empreses, Facultat d'Economia i Empresa, Universitat Rovira i Virgili, 43204 Reus, Tarragona, Spain

**Keywords:** Twitter, Topic modeling, User-generated content (UGC), Construal level theory, Big data, Social media, Social media analytics, Google trends analytics

## Abstract

The COVID-19 pandemic has prompted the re-emergence of staycations to the fore, as many people were forced to spend their vacations at or close to home due to travel restrictions. This phenomenon first went mainstream during the 2008 financial crisis, and has now been further accelerated by the COVID-19 pandemic. This study investigated the growth and practice of staycations during the first two years of the pandemic by analyzing social media and internet search data using Latent Dirichlet Allocation (LDA) topic modeling and Google Trends analytics. Key findings suggest that, while spatially close to home, people tried to achieve a psychological distance away from home. This was demonstrated by a strong global search interest in spending staycations at hotels close to home. The optimal LDA topic model produced 38 topics which were classified under four aggregate dimensions of antecedents, attributes, activities, and consequences of staycations. The findings provide useful insights to managers and policymakers on boosting revenue through this practice, and the role of staycations in promoting leisure activities close to home and sustainable tourism.

## Highlights

• We investigate the practice of staycations during the COVID-19 pandemic from conversations on Twitter and analysis of Internet search trends.
• The LDA topic model produced 38 topics which were classified under four aggregate dimensions.a
• Findings point to a strong interest in spending staycations at hotels to achieve a psychological distance away from home.
• Critical insights are drawn on the role of staycations in promoting tourism closer to home and sustainable tourism.

## 1 Introduction

**Figure 1:**
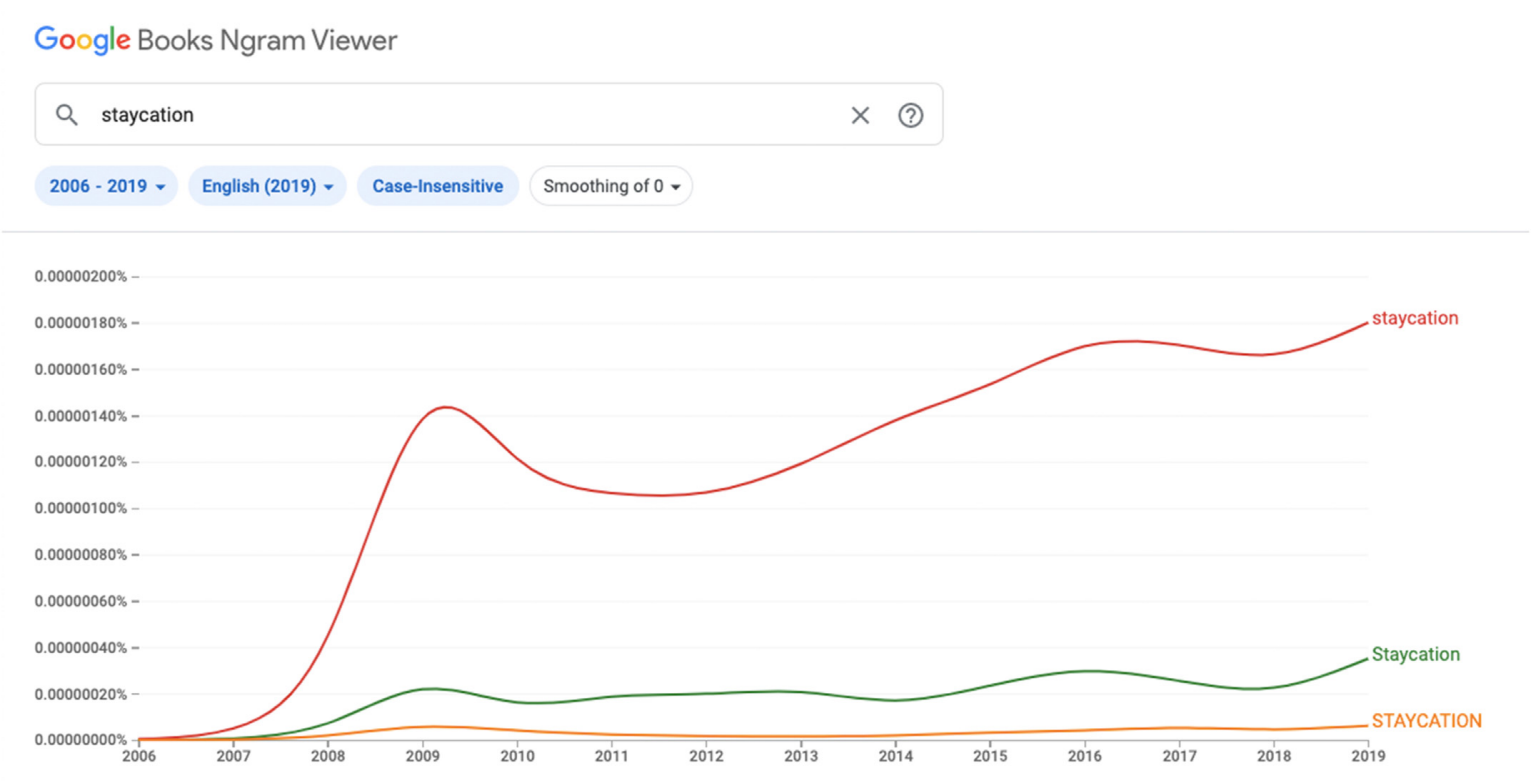
Use of staycation in Google Books Ngram index from 2006 to 2019 (Source: Google Books Ngram Viewer).

International tourism fell dramatically in the aftermath of the 2008 financial crisis as the global economy went into a deep recession with some regions suffering a decline of up to 18% in international arrivals (Smeral, 2010; UNWTO, 2009). As a result of the financial crisis and rising fuel prices, people had less discretionary income to spend on leisure travel and tourism; which led to the substitution of international leisure travel with domestic activities (Papatheodorou et al., 2010). Hence, consumers embraced tourism to places close to home and the word “staycation” received attention in the popular press, entered mainstream usage, and was added to the dictionary during this period (Frew and Winter, 2010; Merriam-Webster,). The evolution of staycation in the Google Books Ngram index shown in Figure 1 equally reflects this. This index contains the text corpora of millions of digitized books and printed sources from 1500 to 2019 in its latest version (Jean-Baptiste et al., 2011). A search in the index for staycation shows that the use of the word was flat until 2006, before growing rapidly and reaching its first peak in 2009 during the financial crisis. Afterwards, its prevalence initially declined slightly, before continuing to grow from 2012 onwards.

Leisure travel and tourism has been hit hard by the COVID-19 pandemic (Gössling et al., 2020). Compared to the global financial crisis, the pandemic has had more devastating consequences for international travel and tourism. At different periods since the beginning of the COVID-19 pandemic, a common tool by various national governments to control the spread of the SARS-CoV-2 virus has been non-pharmaceutical intervention (NPI) measures such as curfews, stay-at-home orders, restriction of public gatherings, lockdowns, travel restrictions, and travel bans with cross-border travel restricted or constrained by tests, quarantine or vaccine requirements (Gössling et al., 2020; Michael Hall et al., 2020). During the early part of the COVID-19 pandemic, international travel came to an almost complete halt with international arrivals plunging by 97% in April 2020 to levels not seen since the early 1990s and resulting in the loss of international tourism revenues by more than ten times those of the financial crisis (UNWTO, 2020). Italy was the first western country to order a nationwide lockdown on March 9, 2020 (Ren, 2020) and by the end of March 2020, over 90% of the world’s population was under some form of international travel restriction resulting in a de facto pause in international leisure travel and tourism (Gössling et al., 2020).

The COVID-19 infection rate in different countries came in waves, and as the first wave of infection subsided with a decline in positive cases around June 2020, some control measures were relaxed that allowed some forms of tourism to resume (Bontempi, 2021; Collins-Kreiner and Ram, 2020). However, research showed that COVID-19 had already inspired a “pandemic travel fear” in people (Zheng et al., 2021). According to a United Kingdom (UK) national survey in August 2020, 68% of respondents cited fears of being stranded abroad and 62% cited uncertainty around COVID-19 as reasons for preferring a staycation in the UK rather than an international holiday (The Cumberland, 2020). After this relaxation of travel restrictions, attempts were made to restart tourism through the promotion of travel to in-country destinations, and domestic tourism showed some signs of recovery in many countries (Michael Hall et al., 2020). Several governments such as in Ireland, Iceland, Italy, Poland, Slovenia, Lithuania, South Korea, Macau, and Thailand started staycation initiatives to encourage visits to local destinations (Cvelbar et al., 2021; Wong et al., 2021).

Hence, staycations have boomed during the COVID-19 pandemic and have been part of the emergent customer behavior inspired by the pandemic (Wong et al., 2021). However, the literature on staycations is still sparse, especially in the context of the COVID-19 pandemic, with most of the studies published after 2008 (de Bloom et al., 2017; Heimtun, 2017; Molz, 2009; Sharma, 2009) and its definition remains unclear. There is confusion about its meaning, especially a conflation with domestic tourism. For example, the British Prime Minister’s summer break in August 2020 in Scotland, hundreds of miles away from his place of usual residence and work in London was widely reported as a “staycation” because it was within the UK (Evening Standard, 2020). Hence, there is a gap in the literature to accurately define and examine the concept of staycations in light of the COVID-19 pandemic and to also evaluate how it has evolved since 2008.

This study analyzes user-generated content (UGC) from Twitter using topic modeling to understand the discourse about staycations on social media and internet search data from Google to study the search interest and behavior about staycations. As a first step, this study explores the identifying elements of staycation and its differentiators from adjacent concepts like domestic tourism, based on the extant academic and industry literature, in order to propose a definition and highlight its benefits. The results of the study are interpreted using Construal Level Theory (CLT), described in the theoretical framework of this study. Finally, the implications of the insights for leisure travel and sustainable tourism are discussed.

## 2 Literature review

### 2.1 Staycation in the literature

Vacations (used interchangeably with holidays in the literature) provide a means for macro-recovery to help people recover from work and everyday stress (de Bloom et al., 2009). Empirical studies have shown that vacations contribute to the quality of life and well-being of many (Dolnicar et al., 2012; Gilbert and Abdullah, 2004). This is besides several other benefits associated with going on vacations. As McCabe (2009b, p. 683) stated: “it is clear that a holiday can have demonstrable impacts connected with many areas of current government policy on: health and well-being particularly in relation to the treatment of stress-related illnesses and disorders but also in a range of other potential treatments or holistic and alternative therapies.” Because of these benefits, going away for a holiday at least once a year has become an integral part of the postmodern society (Urry, 1988). The advent of inexpensive travel amplified this and contributed to the modern consumer culture of mass tourism in which to stay home is to be pitied (Urry, 1988). However, a significant portion of the population in developed economies do not engage in leisure travel (Popp et al., 2021).

Staycation is a portmanteau expression derived from the combination of stay and vacation; and to “take a staycation” means to stay at home during a vacation rather than traveling to a destination, which is presumed to be the point of vacations (West, 2018). Sharma (2009) defined staycations as a neologism referring to “the activity of making a vacation out of staying at home.” Molz (2009) calls it an invented term for describing vacationing at home. Some previous studies credited Terry Massey as the first person to use the word in 2003 (de Bloom et al., 2017; Hay, 2010; James et al., 2017), but the Merriam-Webster dictionary cites a much earlier source in the *Cincinnati Enquirer* newspaper from 1944: “Take a Stay-cation instead of a Va-cation, this year” (Merriam-Webster, n.d.). The use of the word went mainstream during the global financial crisis as soaring fuel prices pushed up airfares, room rates, and other miscellaneous expenses while the global economy was in a recession, which made going for a vacation prohibitively expensive (Fox, 2009; Molz, 2009). Interestingly, the first written usage from 1944 can also be ascribed to a period of crisis as it was a wartime admonition to conserve fuel during the Second World War (Merriam-Webster, n.d.).

**Figure 2:**
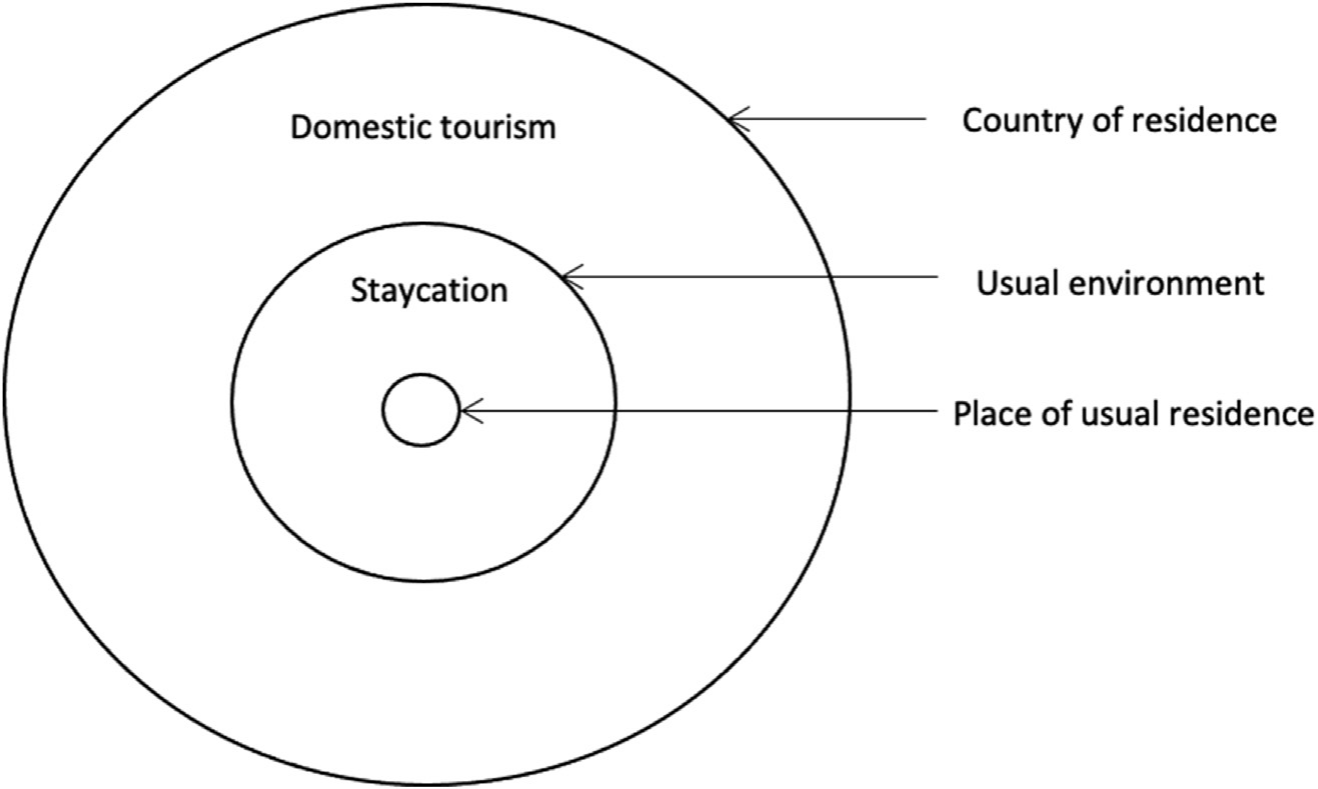
Domestic tourism and staycation

Hay (2010) described staycations as just another name for domestic tourism. However, researchers like de Bloom et al. (2017) distinguished between domestic tourism and staycation and measured the difference in the subjective well-being of a vacation in one’s home or domicile and that of a domestic holiday in their study. This study agrees with the latter and proposes a definition of staycation based on the convention of the World Tourism Organization (UNWTO) for domestic tourism, place of usual residence, and usual environment. The UNWTO defines domestic tourism as activities of a resident visitor within the country as part of a domestic tourism trip or an outbound tourism trip. The place of usual residence as the geographical location a person lives. And the usual environment of a person as the geographical perimeter within which they conduct their regular life routines, which includes their place of usual residence, place of work or study, and all other places visited regularly or frequently, even if far from the usual residence or in another locality, except for vacation homes (UNWTO, 2010). Therefore, staycation could be described as tourism characteristic activities performed within one’s usual environment, as illustrated in Figure 2. Hence, staycation is a subset of domestic tourism, but a domestic tourism trip might not be a staycation if outside one’s usual environment.

### 2.2 Benefits of staycations

People have always spent some of their holidays in their immediate environment without taking a trip for various reasons, but this was not previously considered a vacation or type of tourism (Haukeland, 1990). The novelty is its treatment as a valid type of vacation and the neologism. Researchers like Heimtun (2017) have challenged simplistic notions of tourism and the stereotypical assumption that proper holidays must involve travel in a qualitative study of midlife single women’s home holidays, arguing that it was possible to be a tourist in the home area. Lohmann (1996) found that subjects who traveled during their holidays felt a higher increase in recreation in the middle of their vacation, which fell rapidly after the vacation while the recreation effect of subjects that spent their holiday at home lasted longer after the holidays. In similar findings, de Bloom et al. (2017) found domestic travel to be higher in stimulating engagement in social activity and detachment from work and everyday stress but found no significant difference in the level of pleasure and hedonic well-being generated between domestic travel and a staycation.

Sharma (2009) criticized the manifestation of staycations in the United States (US) during the financial crisis and its gender, class, and race dimensions. In this study, she described this manifestation of staycations as an exercise in waiting out the crisis before real life resumes and everyone got moving again rather than as an opportunity to embrace stillness as an alternative to life in constant motion. Likewise, Molz (2009) also criticized the ambivalent attitude towards staying still in the media coverage and public conversation about staycations in the US during this period as an abnormality to be endured rather than embraced and enjoyed. Staycations have been promoted as beneficial based on advantages due to less travel, cost savings, reduced stress, and a boost to local economies (Frew and Winter, 2010). According to Dodds (2012), staycations like slow travel highlights the opportunities for enjoyable holidays in one’s backyard, which can be as good as that got by traveling for thousands of miles. Finally, a social shift from long to short travel distances from one’s domicile could substantially reduce the greenhouse gas emission contributed by tourism and is more environmentally friendly (Gössling et al., 2010).

### 2.3 Social media and effects of COVID-19 on tourism and hospitality

The literature on the application of social media in tourism and hospitality is extensive and several systematic and bibliometric review studies have been written to summarize the academic contributions in this research area. Social media is important for consumers to acquire information as well as to generate their own content (Zeng and Gerritsen, 2014). These studies have found that the user-generated content (UGC) shared on social media can enable exploration of concepts and meanings linked to tourism and hospitality (Lu et al., 2018). Some of these studies have examined the role of social media as a mega-trend with a significant impact in hospitality and tourism (Leung et al., 2013). While others have focused on how tourism businesses can capitalize on opportunities and tackle challenges presented by social media (Sotiriadis, 2017). Analysis of big data from social media using text mining techniques can also benefit from solid theoretical foundations laid through appropriate social theories (Leung et al., 2017).

The analysis of social media and online communities or conversations has played a critical role in the investigation of the impact of the COVID-19 pandemic on tourism and hospitality, with a huge amount of studies already published. Research using social media has been used to track the emotions of tourism and hospitality employees with data from Reddit (Park et al., 2020), the perception of cruise tourism in the aftermath of COVID-19 outbreaks on cruise ships has been examined using data from Twitter (Muritala et al., 2022), the impact of COVID-19 on Airbnb (Boros et al., 2020), and the use of Instagram by young people unable to travel during lockdown (Dušek and Sagapova, 2022), to mention a few.

### 2.4 Construal level theory

The construal level theory (CLT) is the theory behind the notion of psychological distance. This theory from psychology proposes that human beings only directly experience the present, here, and now. The experience of all other objects beyond the immediate situation is done by forming abstract mental construals of these objects. Hence, psychological distance is a subjective experience that something is close or far away from the self in the present. Psychological distance is egocentric with the self as the reference point, while how an object may be removed from this reference are distance dimensions in time (temporal), space (spatial), social distance, and hypothetical distance (Trope and Liberman, 2010). Research from different domains has converged on the notion that transcending the present requires and is enabled by the human capacity for abstract mental representation (Trope and Liberman, 2010).

CLT postulates that we focus on the abstract at a higher level of construal and on the concrete at lower levels. The perceived distance influences decisions and behavior, i.e. when making choices in psychologically distant situations, people focus on the central or global features of an object (Trope et al., 2007). Several studies in hospitality and tourism have used CLT and there has been a call for more practical applications of CLT in this field (Lindblom et al., 2020). This seminal theory has been employed to investigate how temporal distance and gender influences the assessment of hotel attributes (Kim et al., 2018), the effect of psychological distance or proximity via pictorial information on the evaluation of tourism products (Jia et al., 2021; Marlow and Dabbish, 2014), the relationship between the perception of psychological distance and length of tourist stay (Hateftabar, 2021), the effect of psychological distance on consumer response to destination advertisements (Wang and Lehto, 2020), and the influence of psychological distance on promotional messages in tourism (Kim et al., 2016).

Vacation travel is a beloved pastime and when it was not possible because of the pandemic, people settled for various activities within their usual environment. CLT offers a theoretical foundation for understanding the relationship between psychological distance and these activities and behaviors. Furthermore, previous research has explored COVID-19 leisure activities during lockdown as a way of distancing from the present (Gammon and Ramshaw, 2021). Therefore, this study interpreted findings in light of this theory as people tried to create a simulacrum of their regular vacation while physically at or close to home through the lens of psychological distance. In addition, this theory has not been previously applied to staycations and is a gap in the literature that this study covers.

## 3 Methods

This research consists of two studies, the first is an analysis of Twitter UGC and the second based on analytics of Google Trends data. In the first study, topic modeling of tweets containing staycation was conducted to get the major topics in the data. Topic modeling is a text analytical technique to extract the main features from a body of text that has been applied extensively in the literature, including hospitality and tourism (Muritala et al., 2020; Reyes-Menendez et al., 2020). The tweet IDs were obtained from a published COVID-19 Twitter dataset which covers the year 2020 (Banda et al., 2021). These tweet IDs were rehydrated to get the full tweets using Twarc (Summers et al., 2021). Rehydration is the process by which the complete data about a tweet is requested from the Twitter application programming interface (API). The dataset contained tweets in 65 languages, which we could not translate for our analysis. Therefore, only the tweets in English were rehydrated to save time and computing resources. 103,785,252 full tweets were successfully rehydrated. We loaded the required entries of each tweet with Pandas (McKinney, 2010) into a Python Jupyter Notebook (Kluyver et al., 2016). We then selected the tweets in which staycation appeared and performed data cleaning to reduce the noise in the data by removing irrelevant tweets, i.e. tweets about something else with the staycation hashtag attached. After data cleaning, we carried out pre-processing steps before lemmatizing the data. Lemmatization is a reduction of words to their root word or lemma in order to prepare the text for further processing, e.g. the root of writes, writing, and written is write. The pre-processing steps included removal of stop words (commonly used words like “the”), URL links, white space, newline characters (“/”), distracting single quotes, and other strange quirks present in the text due to the processing of raw text data without the user interface designs of the Twitter platform. After these essential steps, topic modeling was applied to the tweets to identify the major topics or aspects of the online discourse about staycations using Latent Dirichlet Allocation (LDA) implemented using MALLET (version 2.0.8). LDA is an efficient topic modeling technique for extracting the hidden topics from large unlabeled textual data (Blei et al., 2003). MALLET is a Java-based program that uses sophisticated machine learning algorithms to enable faster processing and get a better quality of topic classification (McCallum, 2002).

In the second study, data from Google Trends is analyzed to obtain insights from the search behavior around staycations. Google Trends is an open platform that shows an unbiased sample of anonymized, categorized, and aggregated search queries of a keyword or topic on Google Search, the most popular search engine. It can measure the level of interest in a topic across the globe, in a specific geographical location, or over a specific period (Rogers, 2020). Analysis of internet search query results have been shown to reflect an interest in the real world (D'Avanzo et al., 2017). The data for this study was downloaded using Pytrends (version 4.7.3) (Hogue and DeWilde, 2020), an application programming interface (API) for Google Trends in Python (Van Rossum and Drake, 2009). We obtained the worldwide historical search data for “staycation” from 2004 (when Google started collecting the data) until December 2021, the interest by region, and the “top” and “rising” related search queries and topics for 2020 and 2021, to provide context around the search term. The interest by region shows the relative popularity of staycation searches across different locations. The Top related queries are the most popular terms that users who searched for staycation also searched for. The Rising related queries are terms with the biggest increase in search frequency over the period. The Top related topics are the most popular topics that users who searched for staycation also searched for. While the Rising related topics are topics with the biggest increase in search frequency.

The data for study 1 and study 2 were obtained without applying any geographical filters, hence, they are both presumed to cover the whole world, in order to provide complementary data to spot worldwide trends on staycations and to enhance the validity of this work. In addition, study 2 includes more recent data than study 1, which covers only 2020. This allows us to compare the search trends data between 2020 and 2021, and provides the search trend index up to the time of writing in 2022.

## 4 Findings

### 4.1 Study 1: Topic Modeling

Topic modeling enabled the identification of the major topics in the Twitter UGC to primarily provide insight on the features of COVID-19 staycations and also about new staycation-related activities. 7,729 tweets containing staycation posted by 6,059 unique users were obtained. The mean, median, and standard deviation of tweets per user were 1.276, 1, and 1.929 respectively. This indicates that most of the tweets were probably from personal accounts, and not repeated posts from the same account as typical with news media or blog accounts. Manual examination to identify irrelevant tweets like adverts was carried out, which were then removed (McKinney, 2010). 6,067 tweets were left after data cleaning. Before applying the MALLET algorithm to get the dominant topics in the staycation tweets, we obtain the optimal number of topics for the LDA topic model by calculating the topic coherence score for different numbers of topics and picking the number of topics with the highest topic coherence value. The topic model with the highest coherence score at the end of an upward trend of coherence value or before plateauing usually contains the most meaningful and interpretable topics, hence is usually the optimal model. The topic coherence score of a topic model is a measure of the quality of the topic model (Syed and Spruit, 2017) which computes the sum:

(1) $$Coherence=\underset{i<j}{\sum }score\left(w_{i},w_{j}\right)$$

Eq. (1). Computation of topic coherence score.

**Figure 3:**
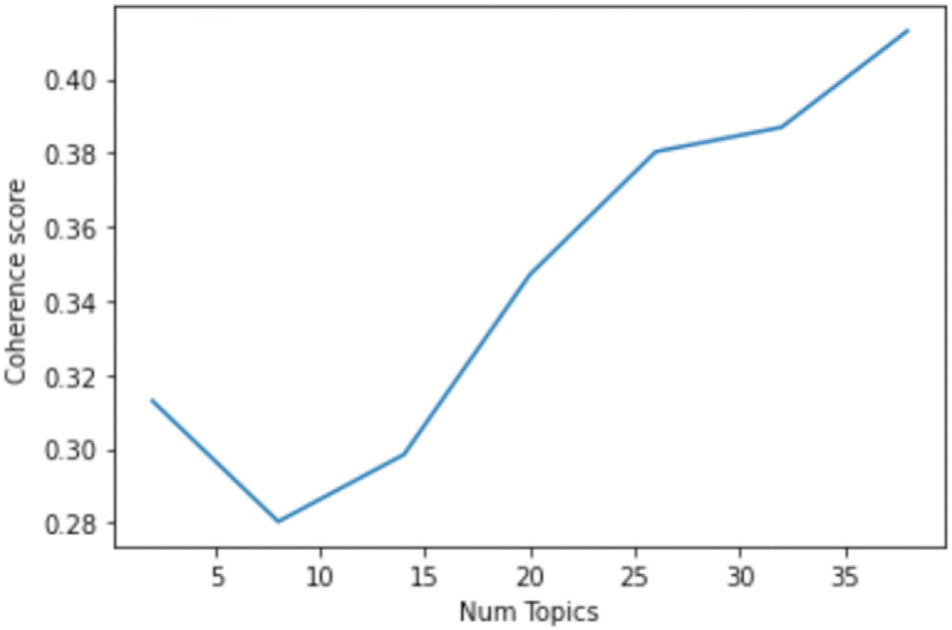
Plot of coherence score against number of topics.

Of the pair-wise scores of words *w*_*1*_*,…, w*_*n*_ used to describe the topic, which are usually the top n words by frequency *p(w|k)* (Pleplé, 2013). After computation, our optimal LDA model was the model with 38 topics with the highest topic coherence score of 0.413 (see Figure 3). The topic keywords, count of tweets, and portion of each topic in the optimum LDA model are presented in Appendix 1.

**Figure 4:**
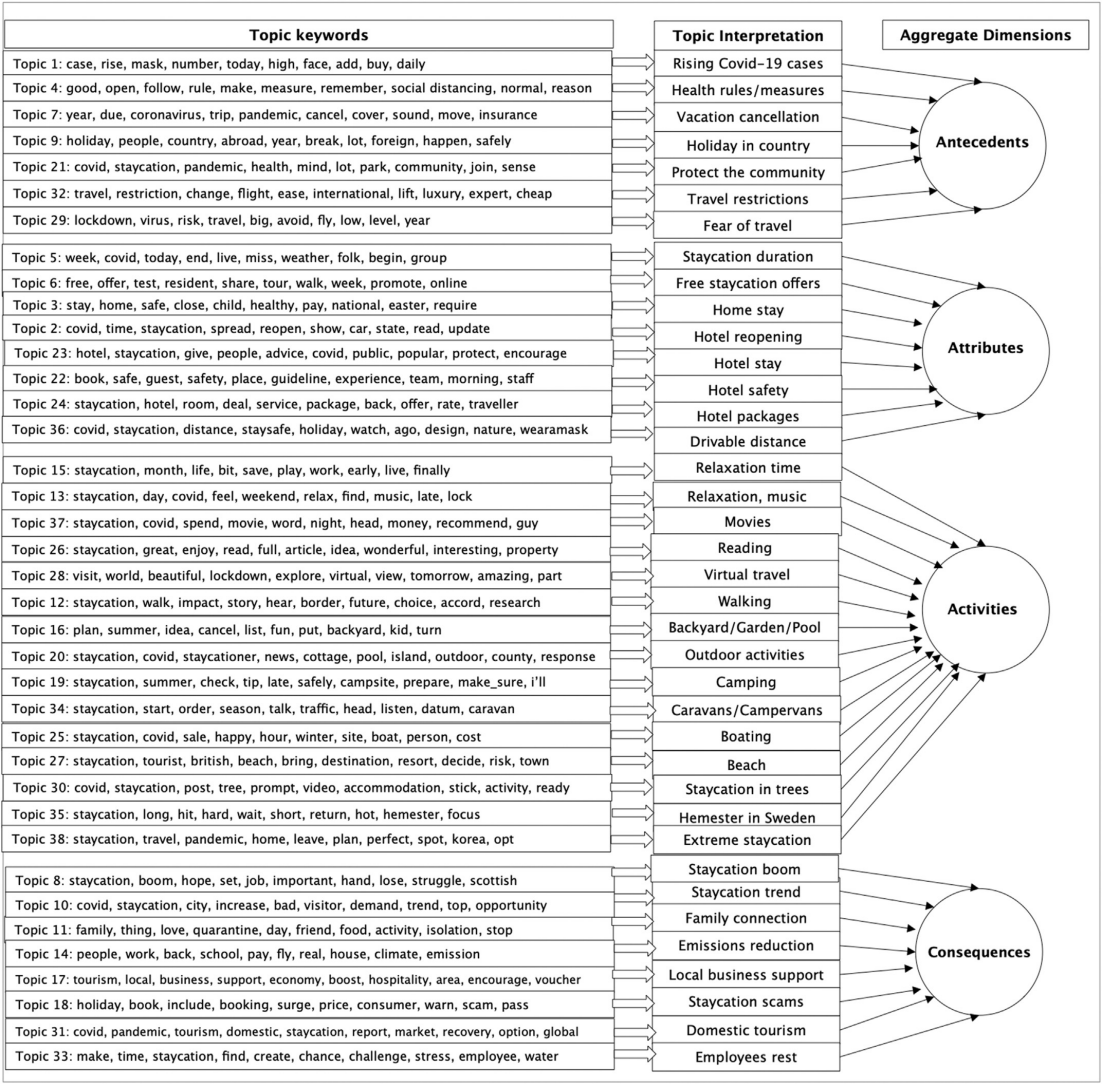
Topic model interpretation and classification.

The interpretation of the topic model is presented in Figure 4. This interpretation was made based on the topic keywords and manual reading of the tweets assigned to each topic. A sample of the tweets under each topic is available in Appendix 2. Furthermore, we grouped the topics into aggregate dimensions in order to generate insights into the broad aspects of the tweets. These aggregate dimensions developed were grouped into the antecedents, attributes, activities, and consequences of staycations. The authors worked separately to group the topics into these aggregate dimensions during a first run, then came to agreement on the classification during a joint second run.

The aggregate dimension on antecedents comprised of tweets on the causes of growth in staycations and is made up of seven topics as follows; the rise in the number of daily cases of COVID-19 (Topic 1), health rules or measures to control the spread of the virus (Topic 4), cancellation of vacation trips (Topic 7), spending holiday in country (Topic 9), protecting the community (Topic 21), travel restrictions (Topic 32), and travel fear (Topic 29).

The aggregate dimension on attributes comprised of tweets on the characteristics of staycations and is made up of eight topics as follows; duration of staycations (Topic 5), staycation deals and hotel staycation packages (Topic 6 & 24), staying home (Topic 3), hotels reopening and encouraging staycations (Topic 2), staying at hotels (Topic 23), hotels mentioning their safety measures (Topic 22), staycation within a drivable distance (Topic 36).

The aggregate dimension on activities comprised of the activities people engaged in and is made up of 15 topics as follows; relaxation (Topics 13 &15), watching movies (Topic 37), reading (Topic 26), virtual travel (Topic 28), walking (Topic 12), backyard activities (Topic16), outdoor activities/cottage stays/swimming pools (Topic 20), camping/caravans/campervans/road trips (Topics 19&34), boating (Topic 25), staying on the beach (Topic 27), Belgians staycationing in tents nested in trees (Topic 30), Hemester (Swedish name for staycation) (Topic 35), “Extreme staycation” in South Korea (Topic 38).

The aggregate dimension on consequences comprised of topics on the consequences of staycations and is made up of eight topics. These include tweets discussing the staycation trend or boom in staycations (Topics 8&10), family connection from staycations (Topic 11), reduction in carbon emissions (Topic 14), support for local businesses (Topic 17), warnings about staycation scams (Topic 18), rise in domestic tourism (Topic 31), and providing employees with an opportunity to rest (Topic 33).

### 4.2 Study 2: Google search trends

**Figure 5:**
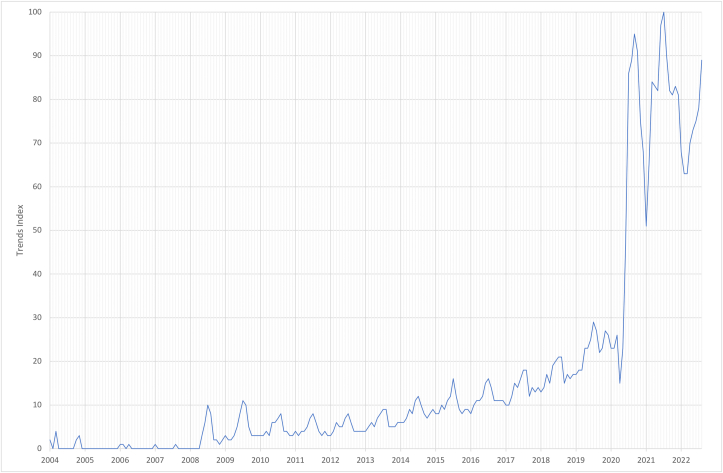
Search interest for staycation from 2004 to 2022 (Data source: (Google Trends, 2022), downloaded on 2022-08-15).

The Google search trends provided insights on the search behavior around staycations on how staycations have changed since 2008 in terms of growth and new activities, as well as the features of COVID-19 staycations. The historical search query for staycation from 2004 till the middle of 2022, showing the search interest in staycations over time, is presented in Figure 5. The data for Figure 5 is normalized with the period of maximum search interest indexed at 100, while all other data points are relative to this. Therefore, an index value of 50 signifies that the term is half as popular relative to the period of peak popularity. While a value of zero means there was not enough data. The figure shows that the frequency of the worldwide search for staycation took off from zero in 2008 during the financial crisis and has grown steadily year on year from 2011, with regular peaks around the middle of each year or summer in the Northern Hemisphere when many people take vacations. The search interest skyrocketed in the middle of 2020 because of the COVID-19 pandemic to over three times the level in 2019. Surprisingly, the search interest grew further by approximately 10% from the peak in 2020 to an all-time high in July 2021 although there were less travel restrictions in 2021 compared to 2020 and international tourism grew 4% in 2021 from 2020 (UNWTO, 2022). So far in 2022, the worldwide search interest in staycation has fallen from the 2021 all-time high, but still at more than double the level in 2019 before the pandemic.

Table 1 shows the top 20 locations where searches for staycation were the most popular. The index values do not indicate the absolute query count but the relative proportion of staycation to all other queries in a location. The values are calculated so that the location with 100 is the place where staycation had the most popularity as a percentage of all searches, so a place with a value of 50 means staycation searches were half as popular as the place ranked first. Therefore, a smaller location where staycation-related queries are 50% of all searches in the location gets double the score of a bigger place where staycation-related queries are 25% of all searches. This explains why relatively smaller places like Hong Kong and Singapore top the list. This means that the larger countries on the list, regardless of a low index score, had a significant amount of staycation-related queries relative to all other search queries. The spread of the locations across different continents shows that searches for staycation were a worldwide trend and not restricted to any region.

**Table 1:** Top locations for staycation searches.

	**Country**	**Index**
1	Hong Kong	100
2	Singapore	86
3	Barbados	77
4	St. Lucia	70
5	Cayman Islands	52
6	Philippines	21
7	United Arab Emirates	14
8	Ireland	6
9	Malaysia	6
10	Qatar	5
11	Indonesia	4
12	France	3
13	United Kingdom	3
14	Kenya	2
15	Canada	2
16	United States	1
17	Australia	1
18	Belgium	1
19	Finland	1
20	Thailand	1

Tables 2, 3, 4, and 5 show the Top and Rising related queries and topics in 2020 and 2021. The Top related queries and topics (Tables 2 and 4) are on a relative scale with the most popular query or topic indexed at 100, while all other queries are relative to this. The Rising related queries and topics (Tables 3 and 5) have seen the biggest increase in search volume during the period and are presented with the percentage increase. Rising queries and topics labeled “Breakout” grew by over 5000% during the period. The dominant languages in these tables are English and Chinese (which is spoken in Hong Kong and Singapore, the top two locations for staycation searches).

**Table 2:** Top related queries in 2020 and 2021.

	**2020 Top Related queries**	**Index**		**2021 Top Related queries**	**Index**
1	Hotel	100	1	hotel staycation	100
2	hotel staycation	100	2	酒店 staycation (hotel)	59
3	singapore staycation	95	3	staycation singapore	57
4	staycation 2020	32	4	staycation 2021	41
5	singapore hotel staycation	28	5	staycation 優惠 (discount)	33
6	staycation 酒店 (hotel)	27	6	uk staycation	30
7	staycation deals	27	7	staycation hong kong	29
8	staycation uk	25	8	klook staycation	25
9	staycation ideas	22	9	staycation hotels	25
10	staycation hong kong	20	10	klook	24
11	Sentosa	19	11	staycation deals	22
12	sentosa staycation	19	12	staycation 香港 (Hong Kong)	19
13	staycation meaning	17	13	staycation meaning	19
14	staycation 優惠 (discount)	17	14	staycation hk	15
15	香港 staycation (Hong Kong)	14	15	hotel staycation singapore	15
16	staycation in singapore	13	16	staycation ideas	14
17	staycation covid	12	17	stay	13
18	singapore staycation deals	12	18	tagaytay staycation	13
19	staycation 中文 (Chinese)	12	19	staycation near me	11
20	staycation dubai	12	20	rosewood staycation	11
21	staycation singapore sentosa	11	21	staycation package	11
22	staycation ireland	11	22	rosewood	10
23	staycation promotion	11	23	sentosa staycation	10
24	staycation singapore 2020	11	24	staycation jakarta	10
25	airbnb	10	25	airbnb staycation	10

**Table 3:** Rising related queries in 2020 and 2021.

	**2020 Rising Related queries**	**Percentage**		**2021 Rising Related queries**	**Percentage**
1	staycation 酒店 (hotel)	Breakout	1	disney staycation cruise	Breakout
2	staycation 優惠 (discount)	Breakout	2	uk cruise staycation	Breakout
3	香港 staycation (Hong Kong)	Breakout	3	disney cruise uk	Breakout
4	staycation covid	Breakout	4	ontario staycation tax credit	Breakout
5	klook	Breakout	5	disney staycation cruise uk	Breakout
6	klook staycation	Breakout	6	staycation 優惠 8 月 (offer August)	Breakout
7	staycation approved hotels	Breakout	7	klook staycation 香港 (Hong Kong)	Breakout
8	staycation uk 2020	Breakout	8	disney hotel staycation	Breakout
9	stb staycation	Breakout	9	wm hotel	Breakout
10	staycation deals singapore 2020	Breakout	10	staycation ireland 2021	Breakout
11	staycation voucher singapore	Breakout	11	klook staycation hk	Breakout
12	murray staycation	Breakout	12	東 涌 喜來登 酒店 staycation (Sheraton Tung Chung Hotel)	Breakout
13	staycation singapore phase 2	Breakout	13	staycation lonavala	Breakout
14	upper house staycation	Breakout	14	the arca staycation	Breakout
15	staycation wales	Breakout	15	hyperair staycation	Breakout
16	staycation during covid	Breakout	16	staycation in hyderabad	Breakout
17	半島 staycation (peninsula)	Breakout	17	staycation 2021	2100%
18	hotels open for staycation	Breakout	18	柏 寧 酒店 staycation (Park Lane Hotel)	2100%
19	hotels approved for staycation	Breakout	19	大 澳 文物 酒店 staycation (Tai O Heritage Hotel)	2100%
20	staycation 意思 (meaning)	Breakout	20	staycation roma	2100%
21	staycation 10 月 (October)	Breakout	21	staycation semarang	2050%
22	staycation rebate	Breakout	22	staycation approved hotels manila	2000%
23	hilton singapore	Breakout	23	staycation uk 2021	1600%
24	rosewood staycation 優惠 (offer)	Breakout	24	staycation cruises	1400%
25	andaz staycation	Breakout	25	staycation tax credit	1100%

**Table 4:** Top related topics in 2020 and 2021.

	**2020 Top Related topics**	**Index**		**2021 Top Related topics**	**Index**
1	Staycation	100	1	Staycation	100
2	Hotel	23	2	Hotel	22
3	Singapore	15	3	Singapore	8
4	Discounts and allowances	8	4	Discounts and allowances	8
5	Hong Kong	6	5	Hong Kong	6
6	Idea	4	6	Klook	3
7	Resort	4	7	Resort	3
8	Sentosa	3	8	Tagaytay	2
9	Swimming pool	3	9	Swimming pool	2
10	Ireland	2	10	Sentosa	2
11	Ireland	2	11	Vacation	1
12	Airbnb	2	12	Buffet	1
13	Vacation	2	13	Cruise ship	1
14	Dubai	2	14	Airbnb	1
15	Paris	2	15	Spa	1
16	Spa	1	16	Jakarta	1
17	Klook	1	17	Ritz-Carlton Hotel Company	1
18	Jakarta	1	18	Rosewood Hotels & Resorts	1
19	Marina Bay Sands Singapore	1	19	Voucher	1
20	Lodging	1	20	Ireland	1
21	Voucher	1	21	Hong Kong Disneyland	1
22	Rosewood Hotels & Resorts	1	22	Disney	1
23	Shangri-La Hotels and Resorts	1	23	Selangor	1
24	Conrad Hong Kong	1	24	Hong Kong Disneyland Hotel	1

**Table 5:** Rising related topics in 2020 and 2021.

	**2020 Rising Related topics**	**Percentage**		**2021 Rising Related topics**	**Percentage**
1	Voucher	Breakout	1	Disney Cruise Line	Breakout
2	Rosewood Hotels & Resorts	Breakout	2	P&O Cruises	Breakout
3	Shangri-La Hotels and Resorts	Breakout	3	Selangor	1600%
4	Conrad Hong Kong	Breakout	4	Cruise ship	1300%
5	Rosewood Hong Kong	Breakout	5	Hong Kong Disneyland	1050%
6	Mandarin Oriental Hotel Group	Breakout	6	Sai Kung	700%
7	Kerry Hotel, Hong Kong	Breakout	7	香港东涌世茂喜来登酒店 (Sheraton Hong Kong Tung Chung Hotel)	650%
8	Resorts World Sentosa	Breakout	8	Goa	550%
9	Singapore Tourism Board	Breakout	9	Disney	500%
10	香港W酒店 (W Hotel Hong Kong)	Breakout	10	Tung Chung	500%
11	Express train	Breakout	11	The Walt Disney Company	450%
12	W Singapore - Sentosa Cove	Breakout	12	Hyatt Regency Hong Kong, Sha Tin	450%
13	Grand Hyatt Hong Kong	Breakout	13	Welsh language	450%
14	RUN HOTEL	Breakout	14	ALVA HOTEL BY ROYAL	400%
15	The Langham	Breakout	15	Hong Kong Gold Coast Hotel	400%
16	Four Seasons Hotel Hong Kong	Breakout	16	Hong Kong Disneyland Hotel	300%
17	The Upper House	Breakout	17	Sheraton Hotels and Resorts	300%
18	Andaz	Breakout	18	Tax credit	300%
19	Hyatt Centric Victoria Harbour Hong Kong	Breakout	19	Royal Caribbean International	300%
20	Sofitel Singapore Sentosa Resort & Spa	Breakout	20	Batangas	250%
21	Capella Singapore	Breakout	21	The Mira Hong Kong	250%
22	The Fullerton Hotel Singapore	Breakout	22	Klook	250%
23	Hyatt Centric	Breakout	23	K11 ARTUS	200%
24	Andaz Singapore - a concept by Hyatt	Breakout	24	Island Shangri-La, Hong Kong	200%
25	Holiday Cottage	Breakout	25	Buffet	200%

The most significant observation from Tables 2, 3, 4, and 5 was the strong search association between staycation and hotels. Hotel and staycation were the top two most popular related queries in both years. Hotel was also the most popular related topic in both years (after a subsequent staycation-related topic search). Users also searched for the names of specific hotels and hotel chains in association with staycation as seen in the Rising related lists in Tables 3 and 5. This shows strong search interest for hotels and other types of hospitality accommodation like resorts and Airbnb to spend staycations. Related searches for staycation deals, packages, discounts, allowances, vouchers, and tax credits show that people were interested in low-cost staycation opportunities. An element of people’s search behavior was to add the name of the location, year, or month while searching on the internet for current staycation opportunities. Comparing both years of the COVID-19 pandemic, the Top lists in Tables 2 and 4 are similar with just the position of the terms changing. However, the Rising lists in Tables 3 and 5 show more difference as there was a rise in searches for “staycation cruises” i.e. cruises that sail close to home to no particular or foreign destination before returning. The increase in search frequency pushed cruise ship into the Top related topics in 2021 on Table 4 and this search for staycation cruises was most popular in Singapore, UK, Hong Kong, and the US.

## 5 Discussion

This study investigated staycations during the COVID-19 pandemic with data from Twitter and Google Trends to provide insights on how staycations have changed since 2008 in terms of growth and new activities and the principal features of staycations during the first two years of the pandemic. The findings indicate that there has been a dramatic increase in internet search interest in staycations worldwide since 2008, including unprecedented growth during the pandemic. The most significant aspect of COVID-19 staycations, as shown by the findings, is a dominant association between staycations and hotels, as demonstrated by four of the topics in the topic model and the internet search analysis. This indicates that many people had staycations at hotels during the pandemic based on the Twitter analysis or were interested in doing so based on the Google search analysis. To interpret this key finding using the Construal Level Theory (CLT), a principal benefit of vacation travel is the provision of psychological distance and freedom from everyday routines and bothersome chores (de Bloom et al., 2017). CLT contends that travel can be construed as freedom from routine, representing a high-level construal (Kim et al., 2016). The function of high-level construals is to enable people to mentally transcend the present by forming a representation of the central features of the object and projecting those representations onto distal situations. Since one of the central features of travel tourism is staying at hotels or other hospitality accommodations, it is easy to build a mental construal of the travel process by staying in a hotel even when spatially close to home. Hence, staycation in a hotel may simulate travel abstractly and help to achieve psychological distance away from home, as everyday routines and chores are avoided while lodging in a hotel, leading to a more positive and satisfactory experience and a higher increase in recreation.

In the same vein, the numerous 5-star hotels and luxury hotel amenities like spa, swimming pool, and buffet in the related search tables which people searched for with staycation indicate a desire for something different by users from what is available at home. All these fit into the mold of people trying to create experiences that can generate high-level construal, as suggested by CLT. Safety measures by the hotels, such as increased cleaning regimes to encourage patronage for staycations, was an important topic in the context of the COVID-19 pandemic. Other studies during the pandemic have shown that these safety measures were of prime importance to attracting customers (Park and Lehto, 2021). The strong connection between staycation and hotels in this study represents a novel finding that was not present in the past literature.

Other key aspects of COVID-19 staycations include staycation-related Google searches for deals, packages, discounts, allowances, vouchers, and tax credits, including two topics in the topic model show people were interested in staycation opportunities at a reduced cost. Benefits related to travel like breaking the routine and enjoying free time are facilitated when they are affordable, making it easy to form a mental construal of travel according to CLT. These offers, deals or discounts can be divided into two; those provided by hotels and those implemented by governments. Hotel managers are encouraged to provide staycation packages for locals, which may compensate for part of the shortfall from international visitors during the ongoing pandemic (García-Gómez et al., 2021). After the pandemic, this practice can remain useful, especially during off-season periods for tourism. Search users were also observed to add the current year, month, or place to their search queries. Hence, adding these time and place markers to the staycation package information on the hotel website is recommended as a good Search Engine Optimization (SEO) tactic, so that the hotel package shows up when users search like this.

Staycation offers through vouchers or tax credits can also be deployed by governments to stimulate demand and encourage people to visit local. Even after the pandemic, these tools remain useful to promote local tourism. These staycation vouchers can be a win-win for all parties as reported in the literature, the government, tourism operators, and the locals, while making the people that redeem such staycation vouchers to spend more money locally (Cvelbar et al., 2021). It could also be a tool to address overtourism by attracting local tourists away from the popular tourist destinations in the country while stimulating tourism demand and opening up areas that are not yet so popular. This does not amount to the government paying for people’s holidays, but a proven strategic investment to promote local tourism. For example, Slovenia’s €350 million staycation voucher scheme was estimated to earn up to 0.47 cents back on every euro spent with estimated multiplicative effects on the economy of up to €0.7 billion (Cvelbar et al., 2021).

Several leisure activities surfaced in the topic model such as camping, boating, walking, relaxation, watching television and movies, visiting the shopping mall, outdoor activities, hiking, road trips, backyard activities, visiting the countryside, eating out at restaurants, visiting local attractions, lounging by swimming pools etc. were similar to the leisure activities reported in previous literature and confirms them (Heimtun, 2017; James et al., 2017; Sharma, 2009). According to CLT, these activities facilitate building a mental image, increase relaxation, and reduce the psychological distance to destinations and are associated with positive attitudes.

Previously unreported leisure activities in the literature connected to staycations surfaced in the findings were virtual travel and staycation cruises. Virtual travel involves visiting unknown places through an online immersion process via the information and videos available on the internet. In more sophisticated implementations; this immersion is carried out through Virtual Reality (VR) headsets (Talwar et al., 2022). The COVID-19 pandemic has been tough for the cruise industry (Muritala et al., 2022). However, some parts of the industry opened up in 2021, as vaccination permitted the resumption of sailing in some places, leading to a breakout search for staycation cruises in 2021 for voyages to nowhere, especially in the UK as shown in the related search tables.

There were also activities or concepts that were unique to some locations in the topics, such as people in Belgium spending their staycations in tents constructed in trees. An activity named “extreme staycation” in South Korea, which involved the transformation of the home into a chosen popular destination like Bali, for example. Hemester is the Swedish word for staycation and it was captured as a topic of its own from tweets about Swedes engaging in staycations. According to CLT, these activities facilitate building a mental image and reduce the psychological distance to destinations and are associated with positive attitudes (Marlow and Dabbish, 2014). These activities are also new findings as they were absent from the past literature on staycations.

Perhaps, the most important topic surfaced under the consequences of staycations is how it can help to reduce the carbon emissions due to leisure travel and tourism. As frequent extreme weather events in different parts of the world continues to highlight the reality of a changing climate (Stott, 2016), the role of tourism, which contributes at least eight percent of global greenhouse gas emissions will be increasingly scrutinized (Bigano et al., 2005; Lenzen et al., 2018). International tourism accounts for a disproportionate contribution to environmental emissions but makes up only 16% of tourism trips (Hall et al., 2013). Flight-shaming is already a growing concept and has been found to be more pronounced against people flying for holidays than for work or to visit family and friends (Doran et al., 2021; Gössling et al., 2019). Furthermore, media reports detail government plans in different countries for green taxes and carbon pricing for flights, which may raise airfares (Abnett, 2021; Gatten and Gill, 2021). Therefore, it is not inconceivable for more people to go on fewer holidays abroad in the near future involving long haul travel for leisure for environmental concerns while embracing staycations and domestic tourism in its stead. Periods of crises have usually steered people towards taking holidays closer to home, such as during the Second World War, the global financial crisis or the current pandemic. In the light of the climate change emergency, which increasingly looks like a chronic crisis that will be confronted over several years, it would not be surprising to have a similar outcome, especially if carbon taxes are integrated into travel costs.

### 5.1 Theoretical and practical implications

This study has made some conceptual and theoretical contributions. First, it has proposed a definition for staycation, which we hope is clearer based on the standing conventions of the UNWTO. This study has also used CLT to explain its findings, demonstrating its applicability and an avenue for further experimental research on CLT and staycations. To the knowledge of the authors, this study is the first to analyze related web search queries and topics data in hospitality and tourism research. Many of the past research in this area using Google Trends data has been on forecasting studies. The interesting findings from this data demonstrate that this is a useful method for acquiring insight from Google-search activity on a global or local scale. Finally, this study also contributes to the literature on the impact of COVID-19 on hospitality and tourism.

Previous research has demonstrated the value of internet-generated data and UGC for hospitality and tourism businesses (Mendes-Filho et al., 2018). From a practical point of view, this study recommends hotel managers to use staycations as an opportunity to boost revenue, provide staycation deals or coupons, and share the details of such deals online with the location and time information specified. They are also recommended to provide information on the cleaning regime in their hotels as a central feature in light of the pandemic. Many governments have offered staycation vouchers and tax credits so far during the COVID-19 pandemic, and research has showed that they are beneficial to all parties concerned. These programs should therefore be sustained and exploited in other countries to support and keep hospitality and tourism businesses afloat through the continuing headwinds caused by the pandemic.

## 6 Conclusion

This study has proposed a definition for staycation based on the standing convention of the UNWTO and extant literature. The most significant finding about the characteristics of the staycations during the COVID-19 pandemic was the uncovering of a dominant interest to spend staycations at hotels, which was interpreted using CLT as people trying to achieve a psychological distance away from home while being spatially close to home. New staycation-related leisure activities, like virtual travel and staycation cruises and specific practices in some locations, were revealed in the findings.

At the time of writing in 2022, cross-border travel in some places remains with COVID-19 related requirements for tests or vaccination. The current high inflation and fuel prices in several OECD countries may also impact on the ability to engage in international leisure travel by some consumers in the near term. Tourism experts have stated that they expect a recovery in international tourism to 2019 levels in 2024 or later (DeMicco et al., 2021; UNWTO, 2022). Research during the pandemic has also reported a desire by people to travel closer to home (ETC, 2022; Lee and Han, 2022). Therefore, while staycations and domestic tourism (Bayih and Singh, 2020) cannot make up for the revenue shortfall from international tourism, it is essential that in-country travel and staycation schemes be harnessed as much as possible to protect tourism operators from bankruptcy before international tourism returns to pre-pandemic levels. In the longer term, concerns about the environmental impact of tourism and climate change and a shift towards more sustainable forms of tourism may also encourage tourism closer to home. The COVID-19 pandemic has accelerated the growth and acceptance of staycations, and while people will continue to desire leisure travel to distant tourist destinations, staycations are not a fad. Hence, it is important for government policy and businesses to adapt to this new reality.

### 6.1 Limitations and future research

This study has the following limitations. Social media data is noisy while containing useful information and it is possible that some valuable information was removed in the process of data cleaning. Social media data might also not be generalizable sometimes and may be biased towards the demographic of people using a particular social media network, hence, it is usually a good idea to complement the data from social media with those from other sources as done by this study. Also, the 38 topics surfaced by the LDA topic model were not the only topics present in the data, but the dominant topics, as the algorithm needs to stop at a particular threshold. If not, it is possible to have an extensive but weak model with many more topics. In order to obtain the staycation-related tweets, a single search term of “staycation” was employed because there are no close synonyms at this point and searching for “home” and “vacation” or “holiday” would have yielded too many false positives. This search process could have missed out on some relevant tweets in which staycation was not explicitly mentioned and this is a limitation of this study. Furthermore, Google does not provide the absolute number of search queries for the Google Trends data, as all the data provided are normalized and relative to the maximum in each category and is therefore not suitable for robust statistical manipulation.

This study has interpreted the findings using CLT, but the method employed cannot directly prove the theory. Future experimental studies that can prove the theory are recommended using the aggregate dimensions of antecedents, attributes, activities, and consequences as research variables. Future studies exploring how to exploit staycations in particular locations to derive more economic benefits in terms of revenues and profits, as well as to produce cultural benefits for citizens increasing their knowledge about their local environment, are recommended to expand the research in this area. Studies into the market segments likely to be interested in staycations, the motivations to engage in staycations in different geographical areas of the world, as well as surveys about people’s staycation preferences, are also recommended.

## Declarations

### Author contribution statement

Babajide Abubakr Muritala; Ana-Beatriz Hernández-Lara; Maria-Victoria Sánchez-Rebull: Conceived and designed the experiments; Performed the experiments; Analyzed and interpreted the data; Contributed reagents, materials, analysis tools or data; Wrote the paper.

### Funding statement

This work was supported by the European Union's Horizon 2020 research and innovation programme under the Marie Skłodowska-Curie grant agreement No. 713679 and from the Universitat Rovira i Virgili (URV).

### Data availability statement

Data included in article/supp. material/referenced in article.

### Declaration of interest’s statement

The authors declare no conflict of interest.

### Additional information

No additional information is available for this paper.
